# Alcohol and Skeletal Muscle in Health and Disease

**DOI:** 10.35946/arcr.v43.1.04

**Published:** 2023-11-02

**Authors:** Liz Simon, Brianna L. Bourgeois, Patricia E. Molina

**Affiliations:** Department of Physiology and Comprehensive Alcohol-HIV/AIDS Research Center, Louisiana State University Health Sciences Center, New Orleans, Louisiana

**Keywords:** alcohol, muscles, skeletal, comorbidity, protein synthesis, proteolysis, metabolism, mitochondria

## Abstract

**PURPOSE:**

Alcohol-related myopathy is one of the earliest alcohol-associated pathological tissue changes that is progressively exacerbated by cumulative long-term alcohol misuse. Acute and chronic alcohol use leads to changes in skeletal muscle mass and function. As discussed in this evidence-based review, alcohol-mediated mechanisms are multifactorial with effects on anabolic and catabolic signaling, mitochondrial bioenergetics, extracellular matrix remodeling, and epigenomic alterations. However, systematic studies are limited, especially regarding the acute effects of alcohol on skeletal muscle.

**SEARCH METHODS:**

This review focuses on peer-reviewed manuscripts published between January 2012 and November 2022 using the search terms “alcohol” or “ethanol” and “skeletal muscle” in MEDLINE, PubMed, and Web of Science using EndNote reference management software.

**SEARCH RESULTS:**

Eligible manuscripts included full-length research papers that discussed acute and chronic effects of alcohol on skeletal muscle mass and function in both clinical and preclinical studies. The review also includes alcohol-mediated skeletal muscle effects in the context of comorbidities. The three databases together yielded 708 manuscripts. Of these, the authors excluded from this review 548 papers that did not have “alcohol” or “muscle” in the title and 64 papers that were duplicates or did not discuss skeletal muscle. Thus, of all the manuscripts considered for this review, 96 are included and 612 are excluded. Additionally, relevant papers published earlier than 2012 are included to provide context to the review.

**DISCUSSION AND CONCLUSIONS:**

Both acute and chronic alcohol use decrease protein synthesis and increase protein degradation. Alcohol also impairs mitochondrial function and extracellular matrix remodeling. However, there is a gap in the literature on the known alcohol-mediated mechanisms, including senescence, role of immune activation, and interorgan communication, on the development of alcohol-related myopathy. With increased life expectancy, changing alcohol use patterns, and increasing frequency of alcohol use among females, current observational studies are needed on the prevalence of alcohol-related myopathy. Additionally, the compounding effects of acute and chronic alcohol use on skeletal muscle with aging or exercise, in response to injury or disuse, and in the context of comorbidities including diabetes and human immunodeficiency virus (HIV), call for further investigation. Though evidence suggests that abstinence or reducing alcohol use can improve muscle mass and function, they are not restored to normal levels. Hence, understanding the pathophysiological mechanisms can help in the design of therapeutic strategies to improve skeletal muscle health.

Alcohol misuse is the most common form of substance misuse and is associated with liver, cardiovascular, and metabolic diseases as well as with infections and cancers.^1^ Although an estimated 20% to 25% of people who drink heavily develop alcohol-related liver disease,^2^ 40% to 60% of people with alcohol misuse have alcohol-related myopathy.^3^ Evidence that alcohol use leads to skeletal muscle (SKM) weakness, even in the absence of neuropathology, was independently documented in the 1800s by James Jackson^4^ and Magnus Huss.^5^ More empirical reports that alcohol or its metabolites could directly or indirectly lead to adaptations of SKM mass and function and that there are differences with acute and chronic alcohol misuse were formulated in the 1950s and 1960s.^6–8^

SKM mass is maintained by the balance of anabolic (protein synthesis) and catabolic (protein breakdown) signaling. Major anabolic stimuli—including amino acids, insulin, insulin-like growth factor 1 (IGF-1)—and mechanical loading promote protein synthesis by converging on the mechanistic/mammalian target of rapamycin (mTOR) signaling pathway (reviewed by Bourgeois et al.^9^ and Steiner et al.^10^). SKM protein breakdown occurs through activation of the ubiquitin proteasome pathway (UPP) and SKM-specific ubiquitin ligases or atrogenes; atrogin-1 (also known as muscle atrophy F-box, or MAFbx) and muscle RING-finger protein-1 (MuRF-1) are often used as markers of UPP activation. The second major protein breakdown pathway is activation of the autophagic-lysosomal system that degrades misfolded proteins by formation of a phagophore followed by engulfing of degraded proteins.^9–12^ Myofibers, their structural components, and the extracellular matrix intricately communicate to maintain SKM structure. Though most adult muscle growth is driven by hypertrophy of existing myofibers, muscle stem cells (satellite cells) contribute to myofiber regeneration, especially in response to injury or atrophy.^13,14^ Finally, being highly dynamic and with high energy demands, SKM relies heavily on mitochondria for bioenergetic demands, redox balance, and programmed death signaling (reviewed by Bourgeois et al.^9^). Evidence suggests that alcohol significantly affects all these major attributes of SKM mass and function, as discussed in this review.

Most current knowledge of the systematic cellular and molecular alterations seen with alcohol-related myopathy is from preclinical rodent studies.^15^ However, the pathophysiological mechanisms of alcohol misuse are complex and are influenced by genetics, sex, lifestyle factors, psychosocial determinants, health comorbidities, and patterns of alcohol use.^16^ Published literature in the 1990s and early 2000s provided epidemiological evidence for the prevalence of alcohol-related myopathy.^17,18^ With the changing patterns of alcohol use,^19,20^ changes in dietary and lifestyle choices,^21^ increase in life expectancy,^22^ and increasing frequency of alcohol misuse among females,^19^ there is a need for recent studies on the prevalence and the disease course of alcohol-related myopathy. Moreover, despite evidence for the high prevalence of alcohol-related myopathy, there is limited literature on its effects on aging, whole body metabolism, response to injury or atrophy, and exercise.

One of the challenges in assessing the effects of alcohol consumption on skeletal muscle and other systems is the sometimes-inconsistent definition of drink sizes and drinking levels, particularly when comparing studies conducted in different countries (see Table 1). The National Institute on Alcohol Abuse and Alcoholism provides information about drinking patterns for adults and defines a standard drink in the United States,^23^ although standard drink definitions sometimes differ in other countries.^24^ The World Health Organization maintains a global database that provides information on several alcohol-related topics, including levels and patterns of alcohol use.^16^

## Search Methods

This review is based on a literature search of three databases—PubMed, MEDLINE (OvidSP), and Web of Science’s Core Collection (Thomas Reuters)—using the EndNote program. The literature search included articles published between January 2012 and November 2022. The search terms used were “ethanol” and “skeletal muscle” as MeSH terms in PubMed and title, abstract, and keywords for MEDLINE and Web of Science.

## Results of the Literature Search

The three databases yielded 708 papers, and titles were screened to include only papers that had the terms alcohol or ethanol, and muscle in the title. With this, 548 manuscripts were excluded. Also excluded were 64 manuscripts that were either duplicates across the three databases or manuscripts that did not discuss SKM. Thus, 96 manuscripts are included in the discussion. Eligible studies were those that included acute or chronic effects of alcohol on SKM mass and function in both clinical and preclinical studies.

This review briefly discusses the salient literature related to alcohol effects on SKM prior to 2012 to provide context. Following this, database search results are organized based on acute and chronic effects of alcohol on SKM metabolic signaling pathways as well as structural and functional adaptations. The review also discusses the effects of alcohol on SKM in the context of some comorbidities, including HIV, pain, cancer, and disuse. Finally, the review discusses gaps in literature and identifies some of the salient future directions that can be pursued.

## Results of the Reviewed Studies

### Overview

The effect of alcohol misuse on SKM mass and function is referred to as acute and chronic alcohol-related myopathy. Acute alcohol-related myopathy presents clinically as breakdown of damaged muscle tissue (rhabdomyolysis)^25^ and is the most frequent cause of nontraumatic rhabdomyolysis.^26^ This can occur even with a single binge-drinking session (blood alcohol concentration 0.08 g/dL), and symptoms generally resolve after 1 to 2 weeks of abstinence.^27^ Alcohol-related rhabdomyolysis predominantly affects muscles of the pelvic and shoulder girdles and is associated with increased circulating levels of creatinine kinase and myoglobin, compartment syndrome particularly of the lower extremities, and, in severe cases, acute renal failure.^25,28^ Evidence also indicates that people who have chronic alcohol-related myopathy can be prone to rhabdomyolysis following an alcohol binge and can show signs of episodic myalgia, muscle weakness, and dark-colored urine.^26^ An estimated 0.5% to 2.0% of people with alcohol misuse present with acute alcohol-related myopathy.^26^ Apart from rhabdomyolysis, acute alcohol affects SKM anabolic protein synthesis pathways^29–34^ and catabolic pathways.^35^

Chronic alcohol-related myopathy (CAM) is the most frequent form of alcohol-related myopathy with an overall prevalence of 2,000 per 100,000 people with alcohol misuse.^17^ Clinical signs associated with CAM are progressive proximal muscle weakness, type II fiber (fast twitch glycolytic fibers) atrophy, pain, and myotonia.^26,36^ Onset of CAM is associated with cumulative lifetime or long-term high-dose alcohol consumption.^17,26^ Thus, clinical manifestations of CAM are seen in older individuals (ages 40 to 60) and are more common in people with other comorbidities, with ~ 50% of people with alcohol-related liver cirrhosis and ~ 80% of people with alcohol-related cardiomyopathy presenting with CAM.^26,37,38^ CAM is characteristically marked by decreased protein synthesis, dysregulation of proteins in the insulin signaling pathway and the mTOR complex 1 (mTORC1) pathway, and dysregulation of myofibrillar and sarcoplasmic proteins.^39–41^ In addition, chronic alcohol intake increases SKM catabolic signaling.^39,42,43^ The effects of chronic alcohol use on SKM mitochondrial function are not clear. Early studies in people with CAM showed a lack of association with mitochondrial energy supply.^44^ However, other studies indicate that chronic alcohol consumption increases SKM glycogen and lipid storage, with megamitochondria and dilated sarcoplasmic reticulum.^40^

These alcohol-associated molecular changes could potentially affect muscle strength. In people with a history of alcohol misuse, mean strength increased from baseline over a 5-year abstinence period, but remained significantly weaker than age-matched controls, with more than half of them still showing histological signs of myopathy.^3^ People who consume a single drink (1 g ethanol/kg body weight) showed a significant decrease in peak strength even 36 or 60 hours post-exercise, indicating that alcohol use accentuates the loss of both dynamic and static strength seen with eccentric exercise.^45^ However, consumption of low-dose alcohol (0.5 g ethanol/kg body weight) after eccentric muscle exercise does not affect muscle force.^46^

Thus, epidemiological and molecular data from seminal work provide evidence that both acute and chronic alcohol use adversely affect SKM and clinically manifest as rhabdomyolysis or weakness of the proximal muscles, respectively. Building on this clinical and preclinical research, and with advances in cellular and molecular assays over the past decade, significant strides have been achieved on elucidating the pathophysiological adaptations that lead to acute and chronic alcohol-related myopathy.

### Acute Effects of Alcohol on SKM

#### Acute alcohol effects on SKM anabolic signaling

**Figure 1:**
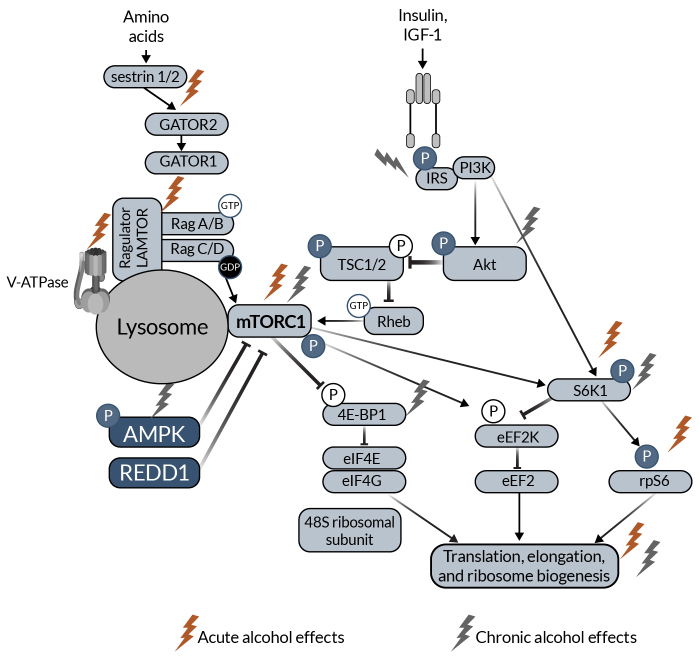
Schematic representation of mTOR anabolic signaling in skeletal muscle The schematic highlights proteins that have been shown to be affected by acute (red lightning bolt icon) and chronic (light gray lightning bolt icon) alcohol intake in the skeletal muscle. *Note:* 4E-BP1, eukaryotic initiation factor 4E-binding protein; Akt, protein kinase B; AMPK, AMP-activated protein kinase; eEF2k, eukaryotic elongation factor 2 kinase; GAP, GTPase activating proteins; GATOR2, toward Rags complex 2; IGF-1, insulin-like growth factor 1; IRS, insulin receptor substrate; LAMTOR, late endosomal/lysosomal adaptor and MAPK and mTORC1 activators; mTORC1, mechanistic/mammalian target of rapamycin complex 1; PI3K, phosphoinositide 3 kinase; REDD1, regulated in development and DNA damage response 1; Rheb, Ras homolog enriched in brain; S6K1, S6 kinase 1; TSC1, tuberous sclerosis complex 1; TSC2, tuberous sclerosis complex 2.

The major SKM anabolic pathway is the mTORC1 pathway leading to muscle protein synthesis (Figure 1). Anabolic stimuli (e.g., insulin, IGF-1) activate phosphoinositide 3 kinase, which phosphorylates and activates protein kinase B (Akt). Akt phosphorylates and inactivates tuberous sclerosis complex 1 and 2 (TSC1, TSC2) through inhibition of TSC2 guanosine-triphosphate hydrolase (GTPase) activity. TSC2 GTPase removes GTP from Ras homolog enriched in brain (Rheb). TSC2 inactivity allows GTP-bound Rheb to accumulate, which stimulates mTORC1 activity. Downstream of mTORC1, S6 kinase 1 (S6K1) is activated, allowing for the activation of ribosomal protein S6. Additionally, eukaryotic initiation factor 4E-binding protein (4E-BP1) is inactivated downstream of mTORC1. Both activation of S6K1 and inactivation of 4E-BP1 increase translational machinery, allowing protein synthesis to increase. Amino acids also regulate the mTORC1 pathway. When amino acids (e.g., leucine) bind to sestrin 1/2, sestrin dissociates from GTPase activating proteins (GAP) toward Rags complex 2 (GATOR2). This decreases the inhibitory effect of GATOR2 on GATOR1. Together, there is a conformational change to Ragulator, which has late endosomal/lysosomal adaptor and MAPK and mTORC1 activators (LAMTOR) increasing GTP-bound RagA and guanosine diphosphate-bound RagC that ultimately activates mTORC1. mTORC1 is also regulated by several other upstream proteins, including AMP-activated protein kinase (AMPK) and regulated in development and DNA damage response 1 (REDD1).^10,47^

In vitro studies in C2C12 myotubes showed that 100 mM ethanol modulates Rag and AMPK/TSC2/Rheb signaling, decreasing the anabolic effects of leucine.^48^ A single intraperitoneal alcohol injection (3 g/kg) in either fasted or fed male mice prevented the increase in fed-state protein synthesis and phosphorylation of ribosomal protein S6 kinase at threonine 389 (S6K1^Thr389^).^49,50^ In the fed state, alcohol administration decreased the association of Raptor and RagC with immunoprecipitated LAMTOR1 and increased sestrin1–GATOR2 and vacuolar-type ATPase V1 association with LAMTOR1 within 1 hour of administration, dysregulating protein–protein interactions of the Rag-Ragulator complex.^50^ However, a study using the same alcohol administration paradigm in REDD1 knockout mice indicated that REDD1 may not play a role in alcohol-mediated decreased protein synthesis, but may be involved in UPP-mediated protein breakdown.^51^

Male mice that were injected with 3 g/kg alcohol intraperitoneally and administered electrically stimulated muscle contractions decreased stimulation-induced total rate of protein synthesis and blunted the phosphorylation of S6K1 (Thr^421^/Ser^424^ and Thr^389^) and its substrate rpS6 (Ser^240/244^), indicating that acute alcohol administration dysregulates stimulation-induced changes in protein synthesis and mTORC1 signaling.^52^ A small clinical study among trained male and female participants who were administered alcohol (1.09 g/kg fat-free body mass) 10 to 20 minutes after an acute heavy resistance exercise trial found decreased exercise-induced phosphorylation of the mTOR(Ser^2448^) and S6K1 (Thr^389^) in males, with no changes in females.^53^ Other studies also showed that alcohol (1.5 g/kg body mass) consumed after exercise decreased mTORC1 signaling and protein synthesis.^54^

Acute alcohol-mediated alterations on the endocrine profile are thought to contribute to impaired SKM anabolic signaling. An alcohol binge post-exercise decreased testosterone to cortisol ratio^55^ or maintained increased testosterone levels post-exercise in healthy young males.^56^ These changes in the hormonal profile can potentially lead to decreased anabolic and increased catabolic signaling, thus affecting SKM protein balance; however, definitive studies are needed to prove this. Moreover, the effects of alcohol on estrogen signaling and their impact on SKM mass and function in both males and females remain largely unknown.

#### Acute alcohol effects on SKM catabolic signaling

A single intraperitoneal injection of alcohol (3 g/kg) blunted the expected fed-state decrease in autophagy in mice, with no significant effects on UPP.^49^ A single intraperitoneal injection of alcohol (5 g/kg) in female mice demonstrated altered expression of genes implicated in fatty acid oxidation, including peroxisome proliferator-activated receptor (PPAR) alpha and PPAR-beta, AMPK, and cluster of differentiation 36 (CD36), as well as of genes involved in protein breakdown, such as MuRF1, Krüppel-like factor 15 (Klf15), and branched chain amino acid transaminase 2 (Bcat2). These changes were associated with increased circulating corticosterone levels and dysregulation of energy substrate metabolism.^57^ C2C12 myoblasts treated with 100 mM of alcohol showed increased proteolysis. An inhibitor of autophagy (3-methyladenine) prevented the increase in proteolysis while a proteasome inhibitor (MG132) did not affect proteolysis, highlighting the relevance of activation of autophagic-lysosomal pathway to alcohol-induced catabolic responses in SKM.^37^

An acute intraperitoneal alcohol injection (5 g/kg) to female mice significantly disrupted mRNA expression of gastrocnemius clock genes as well as clock-controlled genes implicated in SKM function. Alcohol also increased circulating corticosterone levels and one of its target genes, REDD1, in SKM.^58^ The disruption of circadian clocks in different tissues is a possible mechanism of alcohol-induced tissue injury.^59–61^ It remains to be determined whether disruption of the clock genes and circadian rhythm is a mechanism of alcohol-mediated SKM dysfunction and whether there are sex- or age-specific effects.

#### Effects of acute alcohol exposure on SKM function

There are limited studies on the effects of acute alcohol exposure on SKM function. Studies in both male and female mice show that a single intraperitoneal injection of 5 g/kg alcohol decreased absolute and normalized peak-isometric (no change in length) tetanic (continuous muscle contraction) force generated in the triceps surae (gastrocnemius, soleus, and plantaris) muscles and increased fatigue within 1 hour of alcohol administration. These deficits were still present at 24 hours post-alcohol administration in male mice but not in female mice.^62^ In male mice administered 3 g/kg ethanol, there were no differences in twitch or tetanic force in the extensor digitorum longus muscle.^63^ Whether differential kinetics of alcohol clearance or sex hormone differences contribute to the sex-dependent effects warrants further investigation.

In a clinical study from the United States, individuals who consumed alcohol (1.09 g/kg lean mass) had decreased time to exhaustion when using a cycle ergometer 18 to 24 hours post-alcohol consumption, suggesting an alcohol-mediated detrimental impact on severe-intensity exercise performance. However, the single alcohol dose did not affect muscle power, strength, or fatigability (decrease in maximal force in response to contractile activity).^64^ Similarly, heavy episodic drinking (six to 20 or more standard drinks [defined as 8 g of alcohol]) on the previous day in male rugby players in New Zealand decreased lower body performance with no effect on isometric strength and sprint performance.^65^ In Australia, rugby players given 1 g ethanol/kg body weight 4 hours after a rugby match did not have statistically significant differences in maximal voluntary contraction, voluntary activation, or changes in creatine kinase, testosterone, or cortisol the morning after the match.^66^ In the United States, among females who performed two bouts of maximal single leg eccentric extension followed by a single drink (1.09 g/kg fat-free body mass), isometric torque was fully recovered and eccentric torque partially recovered after 48 hours. Using the same exercise regimen, females who consumed a single drink (0.88 g/kg body weight) had no effect on strength recovery.^67,68^ Though the exact mechanisms are not known, it is possible that estrogens play a protective role in muscle recovery in females.^69,70^

One possible explanation for the impaired strength with acute alcohol use is impaired glycolytic function, as severe intensity exercise relies heavily on anaerobic ATP production. In vitro exposure of primary male and female myoblasts to alcohol decreased glycolytic function.^71^ Alternatively, it is possible that the effects of alcohol are indirectly mediated by alterations in endocrine mediators. Acute alcohol binge drinking after resistance exercise increases cortisol levels, a known SKM catabolic mediator.^55,72^ Moreover, alcohol can interfere with SKM regeneration. Normally, muscle-damaging exercises activate regenerative processes with an initial inflammatory response to activate satellite cells.^73^ Acute binge alcohol intake during resistance exercise decreases the early SKM inflammatory response in both trained males and females,^67,74^ providing evidence that it can adversely affect SKM regenerative processes. Moreover, these studies and others have shown marked alcohol-induced decreased myoblast differentiation, suggesting impaired recovery from exercise could be due to decreased regeneration capacity.^75,76^

It should be noted that these effects of single, predetermined alcohol bouts on SKM function were examined in healthy and young males or females; however, whether this holds true in the general population, where the pattern and amount of alcohol consumed is variable, must be considered. Moreover, nutritional state and type of exercise can be confounding factors. In addition, the effect of acute alcohol binges on SKM function during exercise training in people with chronic alcohol misuse also necessitates investigation. In the BEER-High-Intensity Interval Training (BEER-HIIT) study conducted in Spain, females who consumed 12–24 g alcohol per day and males who consumed 24–36 g alcohol per day throughout the 10-week training period did not exhibit lower exercise-mediated increases in lean mass, aerobic fitness, and muscle strength.^77,78^ However, people in treatment for alcohol misuse have decreased isokinetic torque (strength), work, power, and isometric and isotonic muscle loading even after detoxification.^79^ This is compounded by the fact that ~ 15% of people with alcohol misuse have significant mobility impairment.^80–82^

In summary, acute alcohol exposure dysregulates multiple proteins in the mTORC1 pathway and decreases muscle protein synthesis. There is also evidence that acute alcohol use increases catabolic signaling by activating both UPP and autophagy. Acute alcohol intake before or after exercise affects SKM function and is influenced by gender, type of exercise strength, and amount of alcohol. However, the role of other confounders (e.g., age, chronic alcohol misuse) warrants further investigation.

### Chronic Effects of Alcohol on SKM

Studies to identify when CAM develops are difficult and confounded by underreporting of its overall prevalence. A clinical observational study among males from Russia reports that CAM takes about 10 years to develop, with proximal paresis occurring only in people who have muscle atrophy.^83^

Some of the common causes that lead to dysfunctional SKM mass, especially with chronic alcohol use, are increased inflammation and oxidative stress. Zebrafish exposed to 0.5% alcohol for 8 weeks had decreased body weight and muscle fiber cross-sectional area with increased expression of the pro-inflammatory cytokines interleukin 1-beta (IL-1-beta) and tumor necrosis factor alpha (TNF-alpha) and increased expression of high-mobility cassette-1/toll-like receptor 4/nuclear factor-kappa B (HMGB1/TLR4/NF-kappa B) signaling proteins.^84^ Similarly, chronic alcohol-fed rats showed increased SKM TNF-alpha and IL-6 expression and activation of the Janus kinase (JNK) pathway.^85^ Clinical studies also confirmed that TNF-alpha expression was negatively associated with lean muscle mass in people with chronic alcohol misuse.^86^ The increase in inflammation has also been linked to SKM oxidative stress and tissue dysfunction.^87^ Rats with chronic exposure to alcohol had decreased antioxidant enzyme activity and increased malondialdehyde content.^88^ The contribution of oxidative stress to increased protein degradation and SKM dysfunction was demonstrated in C2C12 myoblasts and mitochondria-targeted Mito-TEMPO attenuated alcohol-mediated increase in autophagy.^89^

#### Chronic alcohol effects on SKM anabolic signaling

Chronic alcohol intake upregulates IGF binding protein-1 and myostatin, leading to decreased SKM protein synthesis.^11^ Chronic alcohol feeding of rats for 14 weeks decreased SKM protein synthesis and prevented the anabolic effects of leucine administration irrespective of sex, indicating that there are no sex-specific effects of alcohol on SKM protein synthesis. However, the study also showed that at 6 weeks, males had decreased SKM protein synthesis, but there were no changes in females.^90^ Alcohol administration for 4 weeks in female mice found dephosphorylation of mTORC1 and AMPK, which was mechanistically linked to an increase in protein phosphatase 2A.^91^ Additionally, chronic alcohol administration in mice, ethanol treatment of C2C12 myoblasts, and analyses in people with alcohol-related cirrhosis all demonstrated that alcohol had synergistic effects with increased ammonia to impair SKM protein synthesis and increase protein breakdown.^92^ Multiomics analyses of alcohol-treated C2C12 cells and SKM from ethanol-fed mice identified several beta-hydroxymethyl-butyrate–responsive targets. Moreover, beta-hydroxymethyl-butyrate restored ethanol-induced decreased mTORC1 signaling, protein synthesis, and mitochondrial respiration as well as decreased sarcopenic phenotype.^93^ In a study in Russia, females who reported consuming about 11 units of ethanol per day (1 unit is 10 ml of pure [96%] ethanol) for an average of 5.6 ± 0.6 years had decreased plasma IGF1 levels and decreased SKM expression of insulin receptor substrate (IRS-1), p-AktB, and p-4E-BP1. This was also associated with decrease in cross-sectional fiber area of both type I (slow oxidative) and II (fast glycolytic) fibers.^94^ Similarly, middle-aged males with chronic alcohol misuse had decreased circulating IGF-1 levels and reduced SKM expression of IRS-1 and p-4E-BP1. This was also associated with increased mRNA expression of heat shock proteins and atrogenes and a relative increase in the proportion of fast glycolytic muscle fibers,^95,96^ indicating both a decrease in anabolic signaling and an increase in catabolic signaling.

Studies suggest that chronic alcohol consumption does not affect SKM glucose uptake,^97^ despite the fact that chronic ethanol administration in rats reduced gastrocnemius expression of IRS-1, Akt, and p70S6K.^98^ Chronic alcohol intake increased triglyceride deposition and decreased glucose uptake in SKM, which can lead to metabolic dysregulation.^99^ Additionally, pigs fed a hypercaloric high-fat diet and alcohol diet for 7 weeks showed increased expression of proteins in the insulin signaling pathway and hyperglycemia.^100^ In addition, PPAR-delta activation protected against alcohol-induced decreased Akt phosphorylation and increased mitochondrial uncoupling, indicating that PPAR-delta activation can protect against alcohol-induced SKM lipotoxicity and insulin resistance.^99^

Physical activity or structured exercise generally is beneficial to anabolic signaling. However, female mice that were provided access to running wheels for 5 weeks and access to 20% ethanol in water during the last 5 days had decreased exercise-induced SKM p70S6K phosphorylation and increased MAFbx expression, suggesting that perhaps physical activity alone might not be sufficient to counteract the effects of alcohol-mediated SKM changes.^101^

#### Chronic alcohol effects on SKM catabolic signaling

Zebrafish exposed to 0.5% alcohol for 8 weeks had increased expression of markers of SKM atrophy and autophagy. This was associated with concomitant increases in reactive oxygen species content, decreased mRNA expression of antioxidant enzymes, and protein expression of Nox2,^84^ linking the catabolic changes to increased oxidative stress. Similarly, rats fed 3 g/kg body weight of alcohol for 4 weeks showed increased SKM MuRF1 expression, decreased pAkt/Akt ratio, and increased p-FoxO/FoxO ratio^102^ indicating UPP activation. In contrast, chronic alcohol feeding did not result in increased SKM expression of atrogenes in older rats.^103^ However, there are some discrepancies regarding the activation of SKM autophagy with chronic alcohol misuse. For example, some research showed increased expression of autophagy markers in people with alcohol-related cirrhosis and chronic alcohol-fed mice.^37^ However, this was not observed in other studies of chronic alcohol-fed mice^11^ or in primary myoblasts isolated from in vivo chronic binge alcohol administered to macaques.^76^

#### Chronic alcohol effects on SKM structural characteristics

A clinical study from Brazil showed that people who consumed more than 80 g alcohol per day and followed a sedentary lifestyle had reduced SKM index and phase angle (SKM specific indicator of cellular membrane integrity).^104^ Similarly, in the United States, people with chronic alcohol misuse had significantly lower whole SKM area^37^ and lower femoral and gluteal muscle areas.^105^ However, in a large 12-year observational study among Korean people, high protein intake compared to low protein intake was protective against the development of low SKM mass index. Alcohol consumption in females but not males reduced the protective effect of high protein intake. Among the total participant population who consumed diets with high protein content, heavy drinking was not associated with development of low SKM mass index,^106^ suggesting that dietary and lifestyle modifications potentially can prevent the imbalance in protein turnover with alcohol use.^107^ In an observational study from Russia, females who reported consuming 11 ± 1 units of ethanol/day for an average of 5.6 ± 0.6 years had decreased cross-sectional area of both type I and type II fibers; decreased expression of titin and nebulin, two large proteins involved in maintaining the sarcomere structure; and increased expression of the protease calpain-1 and ubiquitinated proteins.^94^ These findings are consistent with those from preclinical studies showing that chronic alcohol administration for 6 months in rats increased autolysis of mu-calpain; decreased titin, nebulin, and titin hyperphosphorylation; and led to the development of hindlimb muscle atrophy.^108^ Chronic alcohol also decreased expression of myosin heavy chain (MHC), a major motor protein in the thick filament, and troponin-T, which is necessary for myosin and actin positioning.^63^ Adult fish maintained at 0.5% ethanol for 8 weeks showed decreased SKM cross-sectional area, and this was associated with decreased SKM miR-140 expression and increased miR-146a expression. miR-140 targets the Notch signaling pathway, whereas miR-146a targets the Notchantagonist Numb, and these changes in miRNAs are implicated as mechanisms for the observed decreased SKM cross-sectional area.^109^

In addition to the contractile and structural SKM proteins, extracellular matrix (ECM) remodeling plays a critical role in regeneration, anabolic signaling, and mitochondrial function. Adult male rats on an alcohol-containing liquid diet for 24 weeks increased expression of collagens, hydroxyproline, and alpha-smooth muscle actin (marker of myofibroblast activation). This was associated with an increase in other matrisome proteins, including integrin-alpha-5, L-selectin, platelet endothelial cell adhesion molecule (PECAM), secreted protein acidic and rich in cysteine (SPARC), and ADAM metallopeptidase with thrombospondin type 1 motif 2 (ADAMTS2). Alcohol also increased the inflammatory cytokines TNF-alpha, IL-12, and IL-6, and decreased IL-10 mRNA expression,^110^ likely contributing to the observed increase in ECM deposition. Finally, chronic alcohol feeding in rats increased expression of transforming growth factor-beta 1 (TGF-beta) and associated receptors along with downstream signaling components,^111^ as well as expression of matrix metalloproteinase 9,^112^ providing evidence for ECM remodeling and promotion of an SKM profibrotic phenotype.

#### Chronic alcohol effects on SKM functional characteristics

In a longitudinal study among Japanese men and women, alcohol use was positively associated with decreased grip strength, and this association did not change over a 2-year period.^113^ Similarly, in a cross-sectional study among people living in China, men who consumed more than 25 g of alcohol per day had increased risk of low muscle mass and grip strength.^114^ In animal studies, female mice consuming 20% alcohol in water for 40 weeks had decreased grip strength and decreased lean muscle mass, without major neuromuscular junction changes, suggesting that muscle weakness is potentially driven by muscle atrophy.^115^ In other studies, fatigability and alterations in twitch and tetanic tension were seen with chronic alcohol intake.^63^ Evidence from studies using alpha-E83K mutant mice suggested that acute alcohol increased force and that this was due to direct actions of alcohol on the extracellular region of the neuromuscular nicotinic acetylcholine receptor (nAChR).^116^

#### Chronic alcohol effects on mitochondrial function

*Caenorhabditis elegans* exposed to ethanol had decreased expression of mitochondrial fission factor dynamin-related protein 1 (DRP-1), and mitochondrial network fragmentation leading to mitochondrial unfolded protein response, which was mechanistically linked to SKM weakness.^117^ Similarly, rats fed a chronic alcohol diet had decreased levels of mitofusin-1, dysregulation of mitochondrial topoisomerase, and decreased mitochondrial membrane integrity.^118^ Chronic alcohol administration also increased SKM 4-hydroxy-2-nonenal, decreased mitochondrial Complex IV and V activity, and decreased acetylcholinesterase expression, potentially indicating that mitochondrial dyshomeostasis was associated with inhibition of acetylcholinesterase, leading to myofiber atrophy.^98^ In addition, chronic alcohol feeding in the context of high-fat diet for 6 weeks decreased SKM Complex I and III activity, antioxidant activity, as well as increased lipid peroxidation in both male and female mice.^119^ A study using parkin-knockout mice fed an alcohol diet for 12 weeks showed that parkin was critical for alcohol-mediated disruption of mitochondrial complex activity, autophagy/mitophagy balance, and apoptosis.^120^ Finally, ethanol treatment of primary myoblasts isolated from male and female macaques during 5 days of differentiation decreased extracellular acidification rate, an indicator of glycolysis, and increased maximal oxygen consumption rate. These changes were associated with decreased differentiation, suggesting that bioenergetic alterations regulate alcohol-mediated impaired myogenesis.^71^ Despite these indications of alcohol-mediated mitochondrial changes, few systematic studies have focused on mitochondrial bioenergetics and function with acute and chronic alcohol intake.

In summary, chronic alcohol-mediated increases in inflammation and oxidative stress contribute to CAM. Chronic alcohol exposure dysregulates multiple proteins in the mTORC1 signaling pathway and decreases SKM protein synthesis. As seen with acute alcohol exposure, chronic alcohol exposure increases protein breakdown by impacting autophagy and UPP. Preclinical and clinical studies provide evidence for decreased SKM mass with chronic alcohol exposure. Though the exact mechanisms of alcohol’s effect on SKM structural and functional characteristics are not known, dysregulation of contractile proteins, muscle regulatory factors, and ion channels may be implicated.^121^ Evidence also suggests that physical exercise and protein-rich diets can potentially reduce the adverse effects of chronic alcohol intake on SKM.

### Alcohol Effects on SKM in the Context of Comorbidities

#### SIV/HIV

With effective antiretroviral therapy (ART), people with HIV (PWH) have a near-normal life expectancy that has increased the earlier occurrence of age-associated comorbidities, including impaired SKM mass and function as well as frailty. Alcohol misuse is a maladaptive coping behavior among PWH^122^ that can exacerbate HIV-specific effects on SKM. Significant knowledge of the effects of chronic alcohol intake on SKM in the context of HIV has been derived from the rhesus macaque model. Chronic binge alcohol (CBA) administration in ART-naïve macaques infected with simian immunodeficiency virus (SIV), a clinically relevant preclinical model of HIV, increased SKM expression of pro-inflammatory cytokines and decreased antioxidant capacity.^123,124^ CBA produced dysregulation of epigenomic networks, including those of transcriptomic, DNA methylation, and miRNA implicated in ECM remodeling, pro-inflammatory milieu, protein homeostasis, calcium and ion homeostasis, neuromuscular junction signaling, and satellite cell growth and survival, providing evidence for mechanisms leading to CBA-mediated SKM loss at end-stage SIV infection.^125^ ECM remodeling was confirmed by increases in SKM hydroxy proline content and collagen expression, as well as by upregulation of expression of TGF-beta, tissue inhibitor of metalloproteinase (TIMP-1), and matrix metallopeptidase 2 and 9 (MMP2 and 9).^126^ CBA also activated UPP, increasing catabolic signaling.^123^

With effective ART regimens and controlled infection, the prevalence of overt SKM wasting in PWH has significantly decreased, especially during the asymptomatic stage of HIV disease. However, there are cellular and molecular changes that contribute to dysfunctional SKM mass. CBA administration decreased myoblast differentiation, myogenic gene expression, and SKM enriched miRs (myomiR) expression.^127^ Data also suggested that miR-206 targeted Class IIA histone deacetylase (HDAC4), and that an HDAC inhibitor could partially ameliorate CBA-mediated decrease in myoblast differentiation.^128^ Ongoing studies have explored the possibility of extracellular vesicles as mediators of intercellular communication contributing to decreased myoblast differentiation. Results showed significant alterations in expression of myomiRs in extracellular vesicles derived from myotubes formed from myoblasts isolated from CBA animals; however, no significant differences existed in extracellular vesicle size or concentration.^129^ CBA also dysregulated expression of genes implicated in mitochondrial homeostasis (e.g., peroxisome proliferator-activated receptor gamma, coactivator 1 beta [PGC-1b]; PPAR-alpha; estrogen-related receptor alpha; and superoxide dismutase) in SKM of ART-naïve SIV-infected male macaques at end-stage disease.^130^ In animals that were treated with ART and with controlled infection, CBA decreased succinate dehydrogenase activity (complex II of the electron transport chain) in type 1 and type 2 fibers, as well as myoblast maximal oxygen consumption rate. Results also indicated that formoterol, a beta-adrenergic agonist, increased myoblast PGC-1b expression and mitochondrial DNA, and improved maximal oxygen consumption rate,^131^ suggesting that exercise training or increased physical activity may help alleviate alcohol-related myopathy.

In a study among PWH with increased fasting plasma glucose but no diagnosed diabetes, negative indicators of myoblast bioenergetic health (proton leak, nonmitochondrial oxygen consumption rate, and bioenergetic health index) were higher among people with higher scores on the Alcohol Use Disorders Identification Test (AUDIT). This was also associated with increased mitochondrial volume and decreased expression of genes implicated in mitochondrial health.^132^ In the same cohort of PWH, circulating miR-206 was decreased in people with recent alcohol use as indicated by positive phosphatidylethanol (PEth).^133^ In another PWH cohort, body composition had significant modulatory effects on frailty, with higher fat-free mass index, body fat, and body mass index associated with decreased frailty risk. The study also indicated a negative association of frailty with fat-free mass index among people with detectable PEth, indicating that increased SKM mass is protective in PWH with alcohol use.^134^

#### SKM pain

SKM pain is associated with several comorbid conditions, including diabetes and HIV. A study in the United States that examined whether people wanted to drink more alcohol when they experienced SKM pain observed that men had a higher risk than women to self-medicate with alcohol.^135^ Preclinical evidence suggests that chronic alcohol administration in mice promotes SKM mechanical hyperalgesia, and that probiotics can significantly reduce this alcohol-induced SKM mechanical hyperalgesia.^136^ Moreover, in an SKM disuse atrophy model, chronic alcohol feeding of rats for 10 weeks produced mechanical hyperalgesia and associated pain-related neuroadaptations, indicating that chronic alcohol misuse could exacerbate complex pain regional syndrome.^137^

#### Cancer cachexia

The association among alcohol, muscle pain, and muscle functional mass also extends to other comorbidities. In mice injected with melanoma cells, those fed 20% alcohol (weight/volume) in water for 3 months had increased SKM inflammation, apoptosis, and protein degradation. There was also a significant decrease in satellite cell numbers and impaired myogenesis, indicating that alcohol exacerbated cancer-associated cachexia.^138^ In a Lewis lung carcinoma mouse model, chronic alcohol administration decreased expression of proteins in the mTORC1 pathway and increased expression of proteins of both UPP and autophagy, indicating a shift to increased SKM catabolism. Moreover, there was increased phosphorylation of Smad and extracellular signal-regulated kinase signaling proteins as well as increased expression of SKM and circulating myostatin, negative regulators of SKM mass.^139^ These studies suggest that alcohol use can exacerbate cancer-associated cachexia.

### Disuse Atrophy and Injury

Another condition where alcohol can produce detrimental SKM changes is in response to disuse or injury. Alcohol misuse puts people at increased risk for injury and falls due to motor incoordination and peripheral neuropathies.^140,141^ In a model of barium chloride-induced injury of the tibialis anterior muscle, chronic alcohol exposure increased inflammation and fibrosis and decreased the cross-sectional area of regenerated muscle fibers.^103^ In a model of cryoinjury of tibialis anterior muscle, chronic alcohol administration increased inflammation, and low-level laser therapy decreased inflammation and improved recovery.^142^ In a model of SKM disuse, chronic alcohol feeding for 10 weeks in ovariectomized or intact female rats indicated that alcohol dysregulated genes implicated in regeneration and increased TGF-beta expression.^134^ In addition, primary macaque myoblasts isolated from in vivo CBA-administered macaques had decreased differentiation potential and concomitant decrease in myogenic gene expression,^76^ indicating that alcohol could affect SKM regenerative potential. In a model of disuse atrophy, repeated binge alcohol administration activated UPP and decreased protein synthesis.^143^ Based on evidence that alcohol can affect satellite cell regenerative function, exacerbate catabolic signaling, and curb protein synthesis, further studies on the effects of alcohol on muscle injury or disuse atrophy are warranted.

### Alcohol, SKM, and Aging

Alcohol misuse is disproportionately on the rise among older individuals.^20^ Approximately 45% of current drinkers age 60 and older consume more than seven drinks per week, and 25% consume more than 14 drinks per week in the United States.^144^ Alcohol use among older individuals potentially decreases SKM mass and function,^145^ increasing the risk of morbidity.^146–148^ Thus, alcohol-related myopathy potentially can exacerbate age-related declines in SKM mass and function. However, there are few published studies on alcohol’s effects on SKM function in aging, and this warrants attention. Aged female F344 rats fed an alcohol diet for 20 weeks showed decreased lean mass and SKM protein synthesis with dysregulation of multiple proteins in the mTORC1 pathway but with no significant effects on catabolic pathways.^149^ In a large observational study of older males in Japan, alcohol misuse and liver fibrosis led to greater loss of SKM mass^150^ and increased intramuscular adipose tissue accumulation.^151^ A study among postmenopausal women found increased risk for sarcopenia among those with high-risk alcohol use.^152^ Similarly, in another large study among elderly Korean women, binge drinking once or more per week was associated with a higher risk for sarcopenia.^152,153^ However, there are some indications that alcohol consumption may not be associated with sarcopenia in older adults.^154^ The increased aging of the population and the rising frequency of alcohol use in both male and female aged individuals make this an important area in need of further investigation.

## Summary and Recommendations for Future Work

As indicated in this review, although there is a large focus on the effects of both acute and chronic alcohol intake on the mTORC1 pathway and protein synthesis, gaps remain in the literature on alcohol’s effects on mitochondrial bioenergetics, protein degradation, and satellite cell function. Additionally, most of the published clinical and preclinical studies are either observational or descriptive, and future studies that are mechanistic and prove causality are highly warranted. This is particularly relevant with respect to acute effects of alcohol and in the context of exercise training, injuries, atrophy, and aging. There is also a gap in literature on additional alcohol-mediated mechanisms such as epigenomic alterations, role of senescence, effects of immune activation, and circadian signaling that can lead to impaired SKM mass and function. In addition, both clinical and preclinical studies on alcohol-mediated SKM effects in the presence of comorbidities are limited.

Few studies have identified whether the effects of alcohol are directly mediated or the result of alcohol metabolism and generation of metabolites such as acetaldehyde.^37^ Although there is evidence of alcohol directly affecting SKM function, especially in ex vivo systems, it is debatable whether significant alcohol metabolism occurs in SKM. It is possible that acetaldehyde can cause adverse effects in SKM. Similarly, the possibility that alcohol-associated SKM effects result from actions of soluble factors or extracellular vesicles released from distant organs, in a true interorgan communication, remains to be explored.^155,156^

Regarding published clinical studies on alcohol-related myopathy, the lack of objective measures of alcohol use, with few studies including biomarkers of alcohol use, confound the ability to draw conclusions and may explain the sometimes discrepant reports in the literature. Most clinical studies rely on self-report of alcohol use instead of validated questionnaires for assessing alcohol use, making it difficult to draw conclusions and comparisons across populations. With the mission of research and health care institutions, and the community at large, for rigor and reproducibility, it is recommended that peer-reviewed studies use standardized alcohol use questionnaires or biomarkers of alcohol use. For example, AUDIT, timeline follow-back, and lifetime drinking history are established self-report questionnaires that can be used, and PEth is a reliable biomarker of recent alcohol use.^157,158^

Abstinence is the most effective treatment for alcohol-related myopathy. Abstinence results in significant improvement in muscle strength,^3,159^ but fails to reach similar levels to those of age-matched controls. Reducing alcohol use is also a good strategy to improve functional SKM mass.^3,159^ An ideal strategy to improve SKM function in persons with alcohol misuse is to increase physical activity or structured physical exercise while reducing alcohol consumption. The efficacy of exercise-induced beneficial effects on SKM function in subjects with varying levels of alcohol use remains to be determined. Similarly, diet modifications also may help improve SKM function and remain to be systematically studied. Overall, elucidating specific mechanisms and increasing fundamental knowledge of alcohol-mediated effects on SKM can help design therapeutic targets to improve SKM health and overall quality of life.

## Figures and Tables

**Table 1:** Alcohol Consumption: Drink Sizes and Drinking Levels in Select Countries

Defining Drinking Levels				
	United States	Iceland and United Kingdom	China, France, Ireland, and Spain	Austria
Standard drink^23^	0.6 fluid oz or 14 g pure alcohol (12 oz regular beer, 5 oz wine, or 1.5 oz distilled spirits)	8 g pure alcohol	10 g pure alcohol	20 g pure alcohol
Binge drinking^23^	Pattern of drinking that brings blood alcohol concentration to 0.08% or higher in 2 hours (four or more U.S. standard drinks in women; five or more U.S. standard drinks in men)			
Heavy drinking^23^	*Women:* Consuming more than three drinks per day or more than seven drinks per week *Men:* Consuming more than four drinks per day or more than 14 drinks per week			
**World Health Organization: Alcohol Consumption Categories** ^16^				
Abstainer	Consumed no alcohol in lifetime or in past 12 months			
Former drinker	Previously drank alcohol but no consumption in the past 12 months			
Consumer	Consumed alcohol in the past 12 months			
Heavy episodic drinker	Consumed 60 g or more alcohol on at least one occasion in the past 30 days			
